# Association between novel *PLCE1 *variants identified in published esophageal cancer genome-wide association studies and risk of squamous cell carcinoma of the head and neck

**DOI:** 10.1186/1471-2407-11-258

**Published:** 2011-06-20

**Authors:** Hongxia Ma, Li-E Wang, Zhensheng Liu, Erich M Sturgis, Qingyi Wei

**Affiliations:** 1 Department of Epidemiology, The University of Texas M.D. Anderson Cancer Center, Houston, Texas 77030, USA; 2 Departments of Epidemiology and Biostatistics, School of Public Health, Nanjing Medical University, Nanjing 210029, China; 3 Department of Head and Neck Surgery, The University of Texas M.D. Anderson Cancer Center, Houston, Texas 77030, USA

**Keywords:** *PLCE1*, polymorphism, SCCHN, risk, susceptibility

## Abstract

**Background:**

Phospholipase C epsilon 1 (PLCE1) (an effector of Ras) belonging to the phospholipase family plays crucial roles in carcinogenesis and progression of several cancers, including squamous cell carcinoma of the head and neck (SCCHN). A single nucleotide polymorphism (SNP, rs2274223) in *PLCE1 *has been identified as a novel susceptibility locus in genome-wide association studies (GWAS) of esophageal squamous cell carcinoma (ESCC) and gastric cardia adenocarcinoma (GCA) that share similar risk factors with SCCHN. Therefore, we investigated the association between potentially functional SNPs in *PLCE1 *and susceptibility to SCCHN.

**Methods:**

We genotyped three potentially functional SNPs (rs2274223A/G, rs3203713A/G and rs11599672T/G) of *PLCE1 *in 1,098 SCCHN patients and 1,090 controls matched by age and sex in a non-Hispanic white population.

**Results:**

Although none of three SNPs was alone significantly associated with overall risk of SCCHN, their combined effects of risk alleles (rs2274223G, rs3203713G and rs11599672G) were found to be associated with risk of SCCHN in a locus-dose effect manner (*P*_trend _= 0.046), particularly for non-oropharyngeal tumors (*P*_trend _= 0.017); specifically, rs2274223 was associated with a significantly increased risk (AG *vs. *AA: adjusted OR = 1.29, 95% CI = 1.01-1.64; AG/GG *vs. *AA: adjusted OR = 1.30, 95% CI = 1.03-1.64), while rs11599672 was associated with a significantly decreased risk (GG *vs. *TT: adjusted OR = 0.54, 95% CI = 0.34-0.86; TG/GG *vs. *TT: adjusted OR = 0.76, 95% CI = 0.61-0.95).

**Conclusions:**

Our findings suggest that *PLCE1 *variants may have an effect on risk of SCCHN associated with tobacco and alcohol exposure, particularly for those tumors arising at non-oropharyngeal sites. These findings, although need to be validated by larger studies, are consistent with those in esophageal and gastric cancers.

## Background

Squamous cell carcinoma of the head and neck (SCCHN) is the fifth most common cancer worldwide [1], which includes malignancies at the sites of the oral cavity, larynx and pharynx. It was estimated that there were 48,010 new cases and 11,260 deaths of SCCHN in the United States in 2010 [2]. Tobacco smoke and alcohol consumption are the well-recognized risk factors for SCCHN, and more recently human papillomavirus (HPV) is recognized as a major risk factor for the oropharygeal cancer [3]. However, accumulative evidence also shows that genetic factors, including family history and polymorphisms in genes involved in multiple biological pathways, such as carcinogen metabolism, DNA repair, cell cycle regulation, apoptosis and other cellular processes, play important roles in the etiology of SCCHN [4,5]. Although an increasing number of studies on genetic risk factors for SCCHN have been published, the exact genetic basis of susceptibility to SCCHN is still not well defined.

In the past few years, the wave of genome-wide association studies (GWASs) provided a more robust tool to find novel susceptibility loci or genes for cancer susceptibility by using high-throughput genotyping technology to interrogate a large number of tagging polymorphisms in a high density across the whole genome [6]. To date, more than 50 cancer GWASs have been published, including at least 15 different malignant tumors [7], which has greatly improved our understanding of genetic basis of human cancers. However, only one recent GWAS focused on SCCHN risk and identified five variants at 4q21, 12q24 and ADH gene cluster, significantly associated with risk of upper aerodigestive tract cancers (UATC) including SCCHN [8]. It may be a very small proportion of SNPs associated with SCCHN risk because of the strict criteria for the GWAS significance level (*P *= 10^-7 ^or *P *= 10^-8^). Thus, further exploration for the genetic variants that did not reach the GWAS significance level in the development of SCCHN is needed.

In 2010, two large-scale genome-wide association studies [9,10] simultaneously reported that a new and notable low-penetrance susceptibility locus (rs2274223) located in the phospholipase C epsilon 1 gene (*PLCE1*) was strongly associated with risk of esophageal squamous cell carcinoma (ESCC) and gastric cardia adenocarcinoma (GCA) in Chinese population. Rs2274223 is a non-synonymous SNP located in exon 26 of the *PLCE1 *gene, causing the amino acid change from histidine to arginine. Additionally, one study also showed that the positive rates of the PLCE1 protein in ESCC and GCA tissues were significantly higher than that in normal ones, suggesting a biologically plausible role of *PLCE1 *in carcinogenesis of human cancers [9].

The *PLCE1 *gene resides on chromosome 10q23 and encodes phospholipase C epsilon 1 (PLCE1), which belongs to the phospholipase family that catalyzes the hydrolysis of polyphosphoinositides to generate secondary messengers, such as inositol-1,4,5 trisphosphate and diacylglycerol. Therefore, *PLCE1 *is involved in cell growth and differentiation [11]. Studies have reported that PLCE1 functions as an effector of Ras and plays crucial roles in carcinogenesis and progression of several cancers, including cancers of the intestine [12], skin [13], bladder [14], colorectal [15] and head and neck [16]. All these findings further support the biological plausibility that genetic variations, such as single nucleotide polymorphisms (SNPs) in *PLCE1 *that affect the gene expression or protein functions, may affect the risk of some cancers.

Studies have reported that SCCHN shares some similar risk factors with ESCC and GCA, such as tobacco smoke and alcohol consumption [17-19]. Thus, the exciting results in the GWAS of ESCC and GCA, and the strong biological evidence encouraged us to investigate the association between functional SNPs in *PLCE1 *and susceptibility to SCCHN. In the present study, we performed genotyping analyses of three potentially functional SNPs in *PLCE1 *(rs2274223A/G, rs3203713A/G and rs11599672T/G) in non-Hispanic whites and assessed their associations with risk of SCCHN in our ongoing hospital-based case-control study of 1,098 SCCHN cases and 1,090 cancer-free controls matched to cases on age (± 5 years) and sex.

## Methods

### Study populations

Participant recruitment has been described elsewhere [20,21]. In brief, newly-diagnosed SCCHN cancer patients were consecutively recruited from The University of Texas M.D. Anderson Cancer Center between October 1999 and October 2007. All cases were diagnosed with histologically confirmed SCCHN, and there were no age, sex or stage restrictions. Exclusion criteria included second SCCHN primary tumors, primary tumors of the nasopharynx or sinonasal tract, metastasized cancer from other organs, or any histopathologic diagnosis other than SCCHN. Additionally, patients who had received prior surgery (other than diagnostic biopsies), chemotherapy or radiation therapy before recruitment, and any blood transfusion during the preceding 6 months were also excluded.

Cancer-free controls were recruited from those visitors in outpatient clinics at M.D. Anderson Cancer Center, who were not genetically related to the patients or to each other, and were frequency-matched to the cases on age (±5 years), sex and ethnicity. The inclusion criterion for controls was defined as the self-reported absence of prior history of cancer. After the first survey using a short questionnaire to find out whether potential subjects were willing to participate and qualified in this study, a written informed consent was obtained, and a structured questionnaire was administered by trained interviewers to collect demographic data and environmental exposure history, such as age, sex, ethnicity, smoking and alcohol consumption. After interview, approximately 30-ml venous blood sample was collected from each participant. The response rates for cases and controls were approximately 90% and 85%, respectively. In the analysis, a total of 1,098 cases and 1,090 controls that completed the interview and donated a one-time blood sample were included. All subjects were non-Hispanic whites. This study was approved by the Institutional Review Board of M. D. Anderson.

### SNP Selection

Besides the non-synonymous SNP (rs2274223) identified in the published GWAS, we also searched the NCBI dbSNP database (http://www.ncbi.nlm.nih.gov/ build 131) for other common [minor allele frequency (MAF) ≥ 0.05] and potentially functional SNPs located in the 5' near gene, 5'- and 3'-untranslated regions of *PLCE1 *by using a set of tools at the website SNPinfo (http://snpinfo.niehs.nih.gov/) [22]. In addition, the LD analyses were performed to optimize SNP selection. We selected additional two potentially functional SNPs in *PLCE1*: rs3203713, located in the miRNA binding site (3'UTR) and rs11599672, located in the transcription factor binding site (TFBS in 5' near gene). Taken together, we included three SNPs (rs2274223A/G, rs3203713A/G and rs11599672T/G) in *PLCE1 *for the final genotyping.

### Genotyping

**Figure 1:**
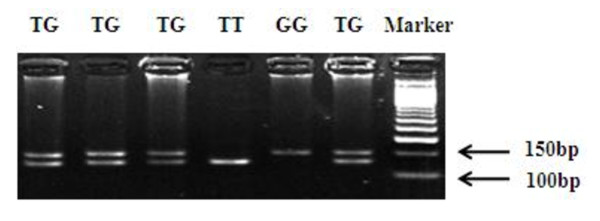
**PCR-based genotyping for rs11599672**.

**Figure 2:**
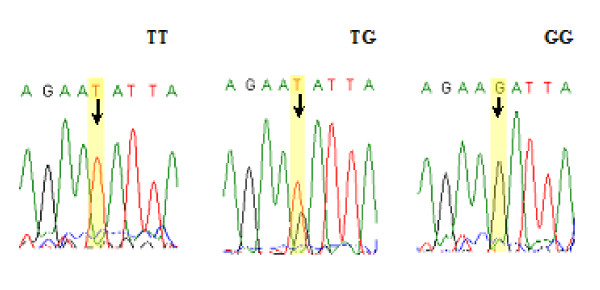
**Direct sequencing results for the *PLCE1 *rs11599672 T > G (TT, TG and GG)**.

We extracted genomic DNA from the buffy-coat fraction of the blood samples using the Qiagen Blood DNA Mini Kit (Qiagen Inc., Valencia, Calif) according to the manufacturer's instruction and genotyped SNPs rs2274223 and rs3203713 using the TaqMan allelic discrimination assay on an ABI 7900 system (Applied Biosystems). Genotyping was performed without knowing the subjects' case or control status, and four negative controls (no DNA) and duplicated commercial positive controls (TaqMan Control Genomic DNA; Applied Biosystems) included in each 384-well plate were used for quality control. The accordance achieved 100% for the duplicates of 5% of samples. Polymerase chain reaction-restriction fragment length polymorphisms (PCR-RFLP) method was also used to identify genotypes of rs11599672, because the Taqman assay was not applicable to this SNP. The antisense primer was introduced a mismatched A to replace T at +2bp from the polymorphic site to create an *Ssp*I restriction site (sense: 5'- GGAGAGACATTCTGTTGGGTGA-3'; antisense: 5'- CCTTCAAACCACCGCTGTA***A***T- 3'). A 155-bp fragment containing this T/G site was amplified in the PCR mixture consisted with approximately 20 ng of genomic DNA, 2 pmol of each primer, 0.1 mM each dNTP, 1 × PCR buffer (50 mM KCl, 10 mM Tris HCl, and 0.1% Triton X-100), 1.5 mM MgCl_**2**_, and 0.5 unit of *Taq *polymerase. The PCR profile consisted of an initial melting step of 95°C for 5 min; 35 cycles of 95°C for 30 s, 59°C for 45 s, and 72°C for 1 min; and final extension step of 72°C for 10 min. The PCR product was then digested with the restriction enzyme *Ssp*I (New England BioLabs, Beverly, MA) overnight at 37°C and separated on 3% agarose gel. The T allele has the restriction site and produces two bands of 134- and 21-bp, whereas the G allele lacks the *Ssp*I restriction site, resulting in one band of 155-bp (Figure 1). Finally, 10% of the samples were randomly selected to perform the repeated assays, and the results were 100% concordant. Direct sequencing was also conducted to confirm the genotypes of rs11599672 (Figure 2).

### Statistical analysis

We evaluated differences in selected demographic variables, risk factors, and frequencies of the *PLCE1 *genotypes between cases and controls by using the χ^2 ^test and examined Hardy-Weinberg equilibrium by a goodness-of-fit χ^2 ^test to compare the observed genotype frequencies with the expected ones among the controls. We estimated associations between *PLCE1 *variants and SCCHN risk by computing odds ratios (ORs) and 95% confidence intervals (CIs) from both univariate and multivariate logistic regression analyses with adjustment for the known risk factors for SCCHN, such as age, sex, smoking status and alcohol use. Haplotype frequencies and individual haplotypes were generated by using SAS PROC HAPLOTYPE based on the observed genotypes. All statistical analyses were two sided, and *P *< 0.05 was considered statistically significant. All the statistical analyses were performed with Statistical Analysis System software (v.9.1 SAS Institute, Cary, NC).

## Results

### Characteristics of study subjects

The characteristics of cases and controls included in this study are summarized in Table 1. The mean age was 57.1 years (±11.1) for cases and 56.6 years (±11.0) for controls. There were no significant differences in the distributions of age and sex between cases and controls (*P *= 0.676 and 0.581, respectively), suggesting the frequency-matching on age and sex was adequate. However, 72.2% and 72.9% of the SCCHN cases were smokers and drinkers, respectively, which were significantly higher than that of the controls (50.9% and 56.6%, respectively) (*P <*0.001 for the both). Of the 1,098 cases, 559 (50.9%) had tumors of the oropharynx and 539 (49.1%) had tumors arising at non-oropharynx sites including oral cavity, hypopharynx and larynx. Furthermore, 272 cases (24.8%) presented with stage I-II and 826 cases (75.2%) presented with III-IV stage.

**Table 1:** Distribution of selected variables in SCCHN cases and cancer-free controls

Variables	Cases No. (%)	Controls No. (%)	***P ***^**a**^
All subjects	1,098 (100%)	1,090 (100%)	
Age, yr			0.676
≤57 (median)	586 (53.4)	572 (52.5)	
>57 (median)	512 (46.6)	518 (47.5)	
Sex			0.581
Females	271 (24.7)	258 (23.7)	
Males	827 (75.3)	832 (76.3)	
Smoking status			<0.001
Never	305 (27.8)	535 (49.1)	
Ever	793 (72.2)	555 (50.9)	
Alcohol status			<0.001
Never	298 (27.1)	473 (43.4)	
Ever	800 (72.9)	617 (56.6)	
Tumor site			
Oropharynx	559 (50.9)		
Non-oropharynx	539 (49.1)		
Stage			
I-II	272 (24.8)		
III-IV	826 (75.2)		

^a ^Two-sided *χ*^*2 *^test.

### Overall associations between PLCE1 variants and risk of SCCHN

The genotype and allele distributions of three SNPs (rs2274223, rs3203713 and rs11599672) in cases and controls are shown in Table 2. The observed genotype frequencies for these three polymorphisms were all in Hardy-Weinberg equilibrium in the controls (*P *= 0.07, 0.98 and 0.72, respectively). The single locus analyses revealed that genotype distributions of these three polymorphisms were not significantly different between overall cases and controls (*P *= 0.554 for rs2274223, *P *= 0.860 for rs3203713 and *P *= 0.265 for rs11599672, respectively). However, we found that the frequencies of variant rs2274223G and rs3203713G alleles (0.335 and 0.170, respectively) among the cases were slightly higher than those in the controls (0.320 and 0.162, respectively), while the frequency of the variant rs11599672G allele among the cases was slightly lower than that among the controls (0.277 *vs. *0.297), suggesting rs2274223G, rs3203713G and rs11599672T alleles may be risk alleles to be considered in further analyses.

**Table 2:** Logistic regression analysis for associations between *PLCE1 *variant genotypes and SCCHN risk

Locus	Genotype	Controls (%)	Overall (N = 1,098)		Oropharynx (N = 559)		Non-oropharynx (N = 539)	
			
		(N = 1,090)	Cases (%)	**OR (95%CI)**^**a**^	Cases (%)	**OR (95%CI)**^**a**^	Cases (%)	**OR (95%CI)**^**a**^
*PLCE1 rs2274223*								
	AA	504 (46.3)	477 (43.5)	1.00	253 (45.3)	1.00	224 (41.6)	1.00
	AG	474 (43.5)	506 (46.1)	1.14 (0.95-1.38)	248 (44.4)	1.09 (0.87-1.36)	258 (48.0)	**1.29 (1.01-1.64)**
	GG	111 (10.2)	114 (10.4)	1.20 (0.88-1.62)	58 (10.3)	1.15 (0.80-1.64)	56 (10.4)	1.38 (0.93-2.06)
	AG/GG	585 (53.7)	620 (56.5)	1.15 (0.97-1.38)	306 (54.7)	1.10 (0.89-1.36)	314 (58.4)	**1.30 (1.03-1.64)**
	G allele	0.320	0.335					

*PLCE1 rs3203713*								
	AA	759 (70.0)	753 (68.8)	1.00	391 (70.1)	1.00	362 (67.4)	1.00
	AG	298 (27.5)	311 (28.4)	1.07 (0.88-1.31)	146 (26.2)	0.98 (0.77-1.25)	165 (30.7)	1.27 (0.99-1.64)
	GG	27 (2.5)	31 (2.8)	1.28 (0.74-2.21)	21 (3.7)	1.74 (0.96-3.16)	10 (1.9)	0.80 (0.36-1.79)
	AG/GG	325 (30.0)	342 (31.2)	1.09 (0.90-1.32)	167 (29.9)	1.04 (0.83-1.31)	175 (32.6)	1.23 (0.96-1.58)
	G allele	0.162	0.170					

*PLCE1 rs11599672*								
	TT	519 (48.3)	554 (50.8)	1.00	270 (48.8)	1.00	284 (52.9)	1.00
	TG	473 (44.0)	469 (43.0)	0.94 (0.78-1.13)	246 (44.5)	1.03 (0.83-1.28)	223 (41.5)	0.81 (0.64-1.02)
	GG	82 (7.7)	67 (6.2)	0.72 (0.50-1.03)	37 (6.7)	0.89 (0.58-1.37)	30 (5.6)	**0.54 (0.34-0.86)**
	TG/GG	555 (51.7)	536 (49.2)	0.91 (0.76-1.08)	283 (51.2)	1.01 (0.82-1.25)	253 (47.1)	**0.76 (0.61-0.95)**
	G allele	0.297	0.277					

Combined effect of risk alleles ^b^								
Trichotomy	0-1	263 (24.6)	235 (21.6)	1.00	124 (22.5)	1.00	111 (20.8)	1.00
	2-3	607 (56.9)	630 (58.0)	1.20 (0.97-1.50)	320 (58.0)	1.14 (0.88-1.48)	310 (58.1)	1.29 (0.97-1.73)
	4-6	197 (18.5)	221 (20.4)	**1.31 (1.00-1.73)**	108 (19.6)	1.23 (0.89-1.70)	113 (21.2)	**1.54 (1.08-2.20)**
				*P *_trend _**= 0.046**		*P *_trend _**= **0.210		*P *_trend _**= 0.017**
Dichotomy	0-1	263 (24.6)	235 (21.6)	1.00	124 (22.5)	1.00	111 (20.8)	1.00
	2-6	804 (75.4)	851 (78.4)	**1.23 (1.00-1.52)**	428 (77.5)	1.16 (0.90-1.49)	423 (79.2)	**1.35 (1.03-1.78)**

^a ^Adjusted for age, sex, smoking and alcohol status. The SNP calling rates were all >98% with 2 samples failed in rs2274223, 9 samples in rs3203713 and 24 samples in rs11599672.

^b ^The risk alleles: rs2274223G, rs3203713G and rs11599672T.

The LD analysis showed that two *PLCE1 *polymorphisms rs2274223 and rs3203713 were in incomplete LD in our study population (*D' *= 0.99, r^2 ^= 0.40), but these two SNPs were not in LD with rs11599672. To estimate possible joint effects and potential locus-locus interactions of *PLCE1 *polymorphisms on risk of SCCHN, we then examined the combined effects of these three variants by the number of putative risk alleles (i.e. rs2274223G, rs3203713G and rs11599672T). As shown in Table 2, we trichotomized the subjects into three groups with "0-1", "2-3" and "4-6" risk alleles. Compared with the group of "0-1" risk allele, there was a locus-dose effect as the risk allele number increased (*P*_trend _= 0.046). After adjustment for age, sex, smoking and alcohol status, the group of " 2-3" risk alleles was borderline associated with risk of SCCHN (adjusted OR = 1.20, 95% CI = 0.97-1.50, adjusted *P *= 0.096) and the "4-6" risk allele group was significantly associated with risk of SCCHN (adjusted OR = 1.31, 95% CI = 1.00-1.73, adjusted *P *= 0.049). When "2-3" and "4-6" groups were combined for a larger number in the same stratum, the association between a larger number of risk alleles and risk of SCCHN remained statistically significant (adjusted OR = 1.23, 95% CI = 1.00-1.52, adjusted *P *= 0.048).

### Associations between *PLCE1 *variants and risk of SCCHN by tumor sites

To investigate the modifying effects of *PLCE1 *variants on risk of SCCHN with different tumor sites, we conducted the stratification analysis by oropharyngeal and non-oropharyngeal cancers. As shown in Table 2, rs2274223 variant genotypes were associated with a significantly increased risk of SCCHN only for non-oropharyngeal sites (AG *vs. *AA: adjusted OR = 1.29, 95% CI = 1.01-1.64, adjusted *P *= 0.042; AG/GG *vs. *AA: adjusted OR = 1.30, 95% CI = 1.03-1.64; adjusted *P *= 0.025), while rs11599672 variant genotypes were associated with a significantly decreased risk of SCCHN for this group of patients (GG *vs. *TT: adjusted OR = 0.54, 95% CI = 0.34-0.86, adjusted *P *= 0.009; TG/GG *vs. *TT: adjusted OR = 0.76, 95% CI = 0.61-0.95, adjusted *P *= 0.018). No association was observed for rs3203713 variant genotypes and risk of SCCHN in both subgroups. In addition, the locus-dose effect of combined risk alleles was also seen in SCCHN arising at non-oropharyngeal sites (*P*_trend _= 0.017). When the group of "0-1" risk allele was used as the reference, the group with "2-6" risk alleles had a significantly higher risk of SCCHN arising at non- oropharyngeal sites (adjusted OR = 1.35, 95% CI = 1.03-1.78; adjusted *P *= 0.033). However, these associations were not found for oropharyngeal cancer.

### Haplotype and stratification analyses

We further evaluated the combined effect of the three polymorphisms on risk of SCCHN arising at non-oropharyngeal sites by using the haplotype analysis (Table 3). A total of six haplotypes were derived from the observed genotypes, of which T_rs11599672_A_rs2274223_A_rs3203713 _was the most common haplotype in cases and controls with the frequencies of 53.6% and 53.0%, respectively. Compared with the common haplotype, the G _rs11599672_A _rs2274223_A _rs3203713 _haplotype was associated with a 28% decreased risk of SCCHN arising at non- oropharyngeal sites (adjusted OR = 0.72, 95% CI = 0.56-0.92); in contract, the T _rs11599672_G _rs2274223_A _rs3203713 _haplotype was associated with a 31% increased risk of SCCHN arising at non-oropharyngeal sites (adjusted OR = 1.31, 95% CI = 1.00-1.72). These associations were not observed for other haplotypes.

**Table 3:** *PLCE1 *haplotype and risk of SCCHN arising at non-oropharyngeal sites

	Haplotype frequencies						
				
***PLCE1 *haplotypes **^**a**^	Cases (N = 1,068)		Controls (N = 2,134)		Crude OR (95% CI)	**Adjusted OR **^**b **^**(95% CI)**	***P ***^**b**^
				
	N	%	N	%			
TAA	572	53.6	1131	53.0	1.00	1.00	
GAA	128	12.0	318	14.9	0.80 (0.63-1.00)	**0.72 (0.56-0.92)**	**0.008**
TGA	119	11.1	198	9.3	1.19 (0.93-1.52)	**1.31 (1.00-1.72)**	**0.049**
GGG	87	8.1	179	8.4	0.96 (0.73-1.27)	0.96 (0.71-1.29)	0.771
TGG	96	9.0	170	8.0	1.12 (0.85-1.46)	1.10 (0.82-1.46)	0.528
GGA	66	6.2	138	6.5	0.95 (0.69-1.29)	0.85 (0.61-1.18)	0.334

^a ^The alleles of haplotypes were arrayed as the location of the SNPs in *PLCE1 *stand from 5' to 3' (e.g. TAA denotes T_rs11599672_A_rs2274223_A_rs3203713_).

^b ^Adjusted for age, gender, smoking and alcohol status in logistic models.

We then performed stratification analyses to evaluate the effects of variant genotypes on risk of SCCHN arising at non-oropharyngeal sites by age, sex, smoking status, alcohol use and disease stage (Table 4). The results showed that the risk effect of rs2274223 was more evident in the younger (adjusted OR = 1.44, 95% CI = 1.02-2.02), male (adjusted OR = 1.51, 95% CI = 1.14-2.00), smokers (adjusted OR = 1.33, 95% CI = 1.02-1.72), drinkers (adjusted OR = 1.69, 95% CI = 1.25-2.28) and subjects with early stage (adjusted OR = 1.40, 95% CI = 1.03-1.91), whereas the protective effect of rs11599672 was more prominent in the younger (adjusted OR = 0.71, 95% CI = 0.50-1.00), smokers (adjusted OR = 0.73, 95% CI = 0.56-0.94) and non-drinkers (adjusted OR = 0.67, 95% CI = 0.46-0.99). Additionally, the increased risk associated with the combined effect of risk alleles was also more pronounced among the younger (adjusted OR = 1.68, 95% CI = 1.09-2.59), male (adjusted OR = 1.41, 95% CI = 1.00-1.98), smokers (adjusted OR = 1.65, 95% CI = 1.21-2.26), drinkers (adjusted OR = 1.42, 95% CI = 1.00-2.03) and subjects with early-stage tumors (adjusted OR = 1.46, 95% CI = 1.01-2.13). However, no significant associations were found for rs3203713 variant genotypes and risk of SCCHN arising at non-oropharyngeal sites in every stratum (data not shown). Heterogeneity test showed that there was no significant heterogeneity (*P *> 0.05) in every two stratums except for that of rs2274223 stratified by alcohol status (*P *= 0.005). Interestingly, we detected a significant interaction between alcohol status (never and ever) and rs2274223 variant genotypes (AA and AG/GG) for risk of SCCHN arising at non-oropharyngeal sites, even after the Bonferroni correction (*P*_int _= 0.004).

**Table 4:** Stratification analysis for associations between *PLCE1 *variant genotypes and risk of SCCHN arising at non-oropharyngeal sites

Variables	rs2274223 (cases/controls)		**Adjusted OR**^**a **^**(95%CI)**	rs11599672 (cases/controls)		**Adjusted OR**^**a **^**(95%CI)**	**Combined effect of risk alleles **^**b **^**(cases/controls)**		**Adjusted OR**^**a **^**(95%CI)**
						
	AA	AG/GG		TT	TG/GG		0-1	2-6	
Age, yr									
≤57(median)	96/286	133/285	**1.44 (1.02-2.02)**	133/284	97/279	**0.71 (0.50-1.00)**	39/140	189/417	**1.68 (1.09-2.59)**
>57(median)	128/218	181/300	1.19 (0.87-1.63)	151/235	156/276	0.88 (0.64-1.20)	72/123	234/387	1.18 (0.82-1.70)
Gender									
Females	84/119	100/139	0.97 (0.64-1.47)	96/110	89/145	0.73 (0.48-1.10)	44/74	140/177	1.30 (0.81-2.08)
Males	140/385	214/446	**1.51 (1.14-2.00)**	188/409	164/410	0.84 (0.64-1.10)	67/189	283/627	**1.41 (1.00-1.98)**
Smoking status									
Never	46/236	57/298	0.99 (0.65-1.53)	54/266	48/261	0.85 (0.55-1.31)	27/116	74/407	0.81 (0.50-1.33)
Ever	178/268	257/287	**1.33 (1.02-1.72)**	230/253	205/294	**0.73 (0.56-0.94)**	84/147	349/397	**1.65 (1.21-2.26)**
Alcohol status									
Never	79/210	76/262	0.84 (0.57-1.23)	86/223	69/244	**0.67 (0.46-0.99)**	34/112	120/352	1.33 (0.83-2.11)
Ever	145/294	238/323	**1.69 (1.25-2.28)**	198/296	184/311	0.86 (0.64-1.15)	77/151	303/452	**1.42 (1.00-2.03)**
Stage									
I-II	87/504	137/585	**1.40 (1.03-1.91)**	114/519	111/555	0.84(0.62-1.14)	44/263	178/804	**1.46 (1.01-2.13)**
III-IV	137/504	177/585	1.24 (0.94-1.65)	170/519	142/555	0.78(0.59-1.03)	67/263	245/804	1.32 (0.94-1.85)

^a ^Adjusted for age, sex, smoking and alcohol status (the stratified factor in each stratum excluded).

^b ^The risk alleles: rs2274223G, rs3203713G and rs11599672T.

## Discussion

In this case-control study, we investigated the associations between three novel, potentially functional SNPs of *PLCE1 *(rs2274223, rs3203713 and rs11599672) and risk of SCCHN in a non-Hispanic white population. Although we did not find evidence of a main effect of each *PLCE1 *SNP on overall SCCHN risk, the joint effect of these variants appeared to contribute to risk of SCCHN in a dose-response manner, especially for cancers arising at non-oropharyngeal sites. Further, the subgroup analysis of SCCHN arising at non-oropharyngeal sites showed that variant genotypes of both rs2274223 and rs11599672 were independently associated with the risk. These findings suggested, for the first time, that potentially functional polymorphisms of *PLCE1 *may play a role in the development of SCCHN, particularly of those tumors at non-oropharyngeal sites.

PLCE1 is known to be a direct downstream effector of small GTPases (Ras, Rap1 and Rap2), with the presence of two Ras-associating domains at its C terminus and a CDC25 guanine exchange factor (CDC25 gef) domain at its N terminus [11,23-25]. Since mutations in the *RAS *gene family are associated with about 30% of all human cancers, several studies have investigated the possible role of PLCE1 in cancer development and progression [13-16,26]. It has been reported that *PLCE1 *has an oncogenic role in carcinogenesis of several human cancers, including SCCHN, through distinct mechanisms, such as inflammation, binding to the Ras family small GTPase, and augmentation of angiogenesis [13-16,26]. Recent evidence has demonstrated that *PLCE1 *mutations might cause the nephritic syndrome [23] and diffuse mesangial sclerosis (DMS) [27]; however, few studies have investigated the association between genetic variants of *PLCE1 *and risk of human cancers.

Most recently, for the first time, Wang *et al. *found that rs2274223, a nonsynonymous SNP of the *PLCE1 *gene, was associated with an increased risk of ESCC and GCA in a Chinese population by a two-stage GWAS in 9,053 ESCC cases, 2,766 GCA cases and 11,013 controls [8]. Likewise, in another GWAS including 2,115 ESCC cases, 2,240 gastric cancer cases and 3,302 controls in another Chinese population, Abnet *et al. *also reported that rs2274223 was a notable signal for susceptibility to ESCC and GCA [10]. Despite the present study with 1,098 SCCHN cases and 1,090 controls may have a limited power to detect weak associations between polymorphisms of *PLCE1 *including rs2274223 and overall risk of SCCHN compared with the published GWA studies [9,10], our results did show that subjects carrying more risk alleles in *PLCE1 *(rs2274223G, rs3203713G and rs11599672T) had a higher risk of SCCHN than those with zero to one risk allele, especially for SCCHN arising at non-oropharyngeal sites, suggesting a joint effect of these SNPs on susceptibility to SCCHN. Given only a modest effect of each SNP individually, evaluating their combined effects may help us better understand any role of *PLCE1 *SNPs in cancer etiology. Furthermore, different carcinogenic mechanisms between esophageal and gastric cardia cancers and SCCHN or genetic difference in different populations may result in the discrepancy for the main effect of rs2274223 between our study on SCCHN and two GWASs of esophageal and gastric cancers.

Studies have shown that the oropharynx is the most common site for HPV-associated SCCHN [28,29]; in contrast, SCCHN arising at non-oropharyngeal sites have much lower HPV seroprevalence [28,30] and thus are more likely to be caused by smoking and alcohol drinking, similar to ESCC and GCA. In fact, 36% of our oropharygeal cancer patients were never smokers compared to only 19% of patients with SCCHN arising at non-oropharygeal sites. In our study, we found that rs2274223 and rs11599672 had an independent effect on risk of smoking and drinking related SCCHN arising at non-oropharyngeal sites but not HPV-related oropharyngeal cancer. Even after Bonferroni corrections, the association of rs11599672 with non-oropharyngeal SCCHN and association of rs2274223 with drinking at non-orpharyngeal sites remained significant, suggesting different roles of these polymorphisms in the etiology of two different tumor subsites. Additionally, subgroup analyses restricted to oropharygeal cancer did find a similar pattern of risk (though non-significant) associated with variant genotypes of rs2274223, rs11599672 and combined risk alleles among smokers but not among never smokers (data not shown). The findings from GWASs and our study implied that polymorphisms of *PLCE1 *are likely to be associated with the development of human cancers related to tobacco and alcohol exposure, which will need the validation from large studies on different cancers.

Further, compared to the most common T _rs11599672_A _rs2274223_A _rs3203713 _haplotype, both T_rs11599672_G _rs2274223_A _rs3203713 _and G _rs11599672_A _rs2274223_A _rs3203713 _haplotypes had a significant association with risk of SCCHN arising at non-oropharyngeal sites, which further reflected the main effect of rs2274223 G and rs11599672 G alleles on risk of SCCHN arising at non-oropharyngeal sites. We also found that the effects of rs2274223 and rs11599672 on risk of SCCHN arising at non-oropharyngeal sites were segregated between the subgroups by smoking or drinking status, indicating the possibility of gene-environment interactions. Indeed, we detected a significant interaction between variant genotypes of rs2274223 and alcohol status with a relatively small sample size, supporting the role of a gene-environment interaction in the development of SCCHN.

It has been identified that rs2274223 is a non-synonymous SNP of *PLCE1*, and bioinformatics tools show that rs3203713 in *PLCE1 *is located in the 3'UTR (http://snpinfo.niehs.nih.gov/snpfunc.htm), which may affect the binding of miRNA and target gene *PLCE1*. Further, rs11599672 is located in the transcription factor binding site (TFBS) of *PLCE1 *(http://snpinfo.niehs.nih.gov/snpfunc.htm) and may result in the variation of transcription activity and expression of *PLCE1*. Considering the potentially functional significance of these SNPs, it is biologically plausible that *PLCE1 *polymorphisms may contribute to the development of SCCHN. However, we cannot exclude the possibility that the findings from a subgroup analysis could be false positive because of a limited sample size. More rigorous studies with larger sample sizes, detailed HPV data and SNP functional relevance are warranted to replicate our findings and identify the underlying mechanism(s) of these SNPs in the etiology of SCCHN.

Some limitations of this study need to be addressed. Firstly, it is a hospital-based case-control study, and inherent selection bias cannot be completely excluded. However, the agreement of observed genotype distributions with Hardy-Weinberg equilibrium and similar allele frequencies of our controls with those reported in CEU populations from the dbSNP database (0.320 *vs. *0.314 for rs2274223 G allele, 0.162 *vs. *0.120 for rs3203713 G allele, and 0.297 *vs. *0.347 for rs11599672, respectively) suggested that selection bias in terms of genotype distribution would not be substantial, if any. Secondly, it is uncertain whether the results from other populations such as Chinese populations in the reported GWASs [9,10] are generalizable to our study population; yet similar findings from our non-Hispanic white subjects further support a similar biological plausibility of these SNPs. Thirdly, while we explored associations with smoking and drinking exposure, we were not able to stratify our data by HPV exposure. Finally, 3 SNPs were included in this study and we cannot rule out the possibility of false-positive associations because of multiple tests. Actually, only *P *values for the effect of rs11599672 variant genotypes on tumors arising at non-oropharyngeal sites and the association of rs2274223 variant genotypes with drinking for non-oropharyngeal SCCHN risk remained significant after Bonferroni corrections.

## Conclusions

In summary, in this hospital-based case-control study, we found that three *PLCE1 *SNPs may have a joint effect on the risk of SCCHN, especially those arising at non-oropharyngeal sites, including oral cavity, hypopharynx or larynx, associated with smoking and alcohol exposure. Furthermore, those results from subgroup analysis suggest that variant genotypes of rs2274223 and rs11599672 may independently affect the risk of SCCHN arising at non-oropharyngeal sites. Additional larger studies in different populations are needed to validate our findings.

## Abbreviations

OR: odds ratios; CI: confidence interval; *PLCE1*: phospholipase C epsilon 1 gene; GWAS: genome-wide association study; LD: linkage disequilibrium; MAF: minor allele frequency; PCR: polymerase chain reaction; SCCHN: squamous cell carcinoma of the head and neck; TFBS: transcription factor binding site

## Competing interests

The authors declare that they have no competing interests.

## Authors' contributions

HXM, LEW and QYW designed the study and wrote the manuscript. HXM performed the experiments with the assistance from ZSL. EMS helped to revise the manuscript. All authors read and approved the final manuscript.

## Pre-publication history

The pre-publication history for this paper can be accessed here:

http://www.biomedcentral.com/1471-2407/11/258/prepub
